# Supplementary material for: Regulatory T Cells Induce Metastasis by Activating Tgf-Β and Enhancing the Epithelial–Mesenchymal Transition
Source: Cells. 2019 Nov 4;8(11):1387. doi: 10.3390/cells8111387 (PMC6912455; doi:10.3390/cells8111387)
Supplement: Supplementary file 1 [file cells-08-01387-s001.pdf]

Supplementary Material

Supplementary Figure 1.

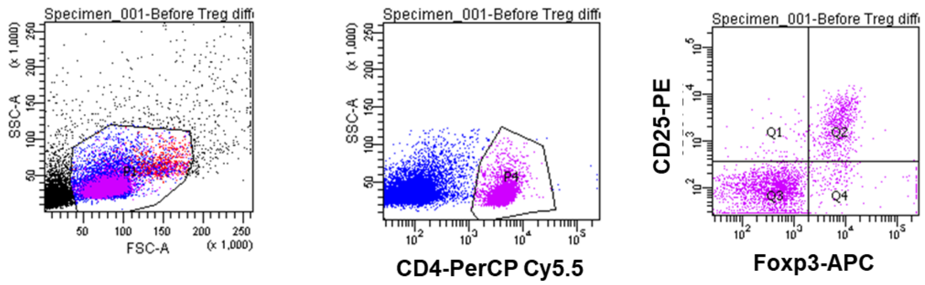

Supplementary Figure S1. Flow cytometry analysis of draining lymph node (DLN) of GFP transgenic mice. Cells from DLN (DLNC) were harvested and analyzed by flow cytometry before Treg differentiation. Gating was for cluster of differentiation (CD)4$^+$ T cells and analysis for CD25$^+$ and Foxp3$^+$ cells.
